# Healthcare workers' views on the response to COVID-19 in long-term care hospitals in Korea: a mixed-method study

**DOI:** 10.3389/fpubh.2025.1518998

**Published:** 2025-06-16

**Authors:** Eun Kyoung Yun, In Seo La, Sunmi Kim, Seongyu Han, Hyungran Lee

**Affiliations:** ^1^College of Nursing Science, Kyung Hee University, Seoul, Republic of Korea; ^2^East-West Nursing Research Institute, Kyung Hee University, Seoul, Republic of Korea; ^3^Department of Nursing, Graduate School, Kyung Hee University, Seoul, Republic of Korea

**Keywords:** long-term care, COVID-19 response, mixed methods, business continuity planning, healthcare workers, infection prevention and control, disaster preparedness

## Abstract

**Introduction:**

Long-term care hospitals (LTCHs) faced challenges beyond the scope of their previous practice in response to the pandemic. However, not much is known about LTCHs' responses and business continuity plans (BCPs) during the pandemic. We investigated attempts by LTCHs to maintain continuity of operation during COVID-19 in order to gain insight on how to support them in future crises.

**Methods:**

A mixed-method design was used, comprising a survey and individual interviews, to understand the responses and measures taken to address the pandemic.

**Results:**

For LTCHs, inpatient ward operations were identified as an essential function. Following the government's recommendation, most (85.7%) confirmed having BCPs, but over half felt that the operational effectiveness of BCPs was inadequate. Only 9.5% formed teams dedicated to infectious disease emergency preparedness and response before COVID-19. Qualitative analysis identified six main themes that explained the efforts of the LTCHs: workplace culture and leadership, communication, human resources, safety, continuity of essential services, and financial and supply management. The themes explained the reasons for operational effectiveness and provided examples and context on how staff responded in small and medium-sized LTCHs during the pandemic, considering elements in health service continuity planning.

**Conclusion:**

Management of significant changes forced by the pandemic necessitates preparing a response that considers key components beforehand, particularly for vulnerable healthcare facilities. To address unexpected crises, LTCHs should develop, implement, and practice well-thought-out plans to enhance organizational resiliency and ensure continued hospital functioning.

## 1 Introduction

The COVID-19 pandemic has posed unprecedented challenges to healthcare systems worldwide, with long-term care facilities being among the most critically affected (1). In long-term care settings, such an infection can rapidly spread by human-to-human transmission through direct contact or droplets (2, 3). As major long-term care facilities, long-term care hospitals (LTCHs) in Korea are hospitals that provide medical services to patients requiring extended care, primarily those with advanced age and a range of chronic underlying conditions (4, 5). These long-term care recipients are highly susceptible to the emerging infectious disease; 94.7% of LTCHs reported experiencing an outbreak in 2022 (6, 7). LTCHs across Korea have become focal points for COVID-19 outbreaks, reporting higher fatality rates (12%) than the domestic COVID-19 fatality rate (1.75%) during the first year since the initial case in Korea (8).

Given LTCHs' significant role in this era of aging, it is necessary to ensure that LTCHs respond appropriately when faced with various risks pertaining to calamities, such as infectious disease outbreaks (9). Managing the spread of the COVID-19 and changes within LTCHs entailed complex challenges that extended beyond standard healthcare practices, including testing all contacts and setting cohorts (10–12). According to an existing review (13) and the related literature (14), some actions taken to adapt to external/internal demand during the pandemic by resilient hospitals were beneficial based on preparedness, whereas measures implemented by other hospitals revealed vulnerabilities.

As such, the pandemic has highlighted the importance of building hospital resilience. Hospital resilience refers to a system's capacity to promptly and effectively withstand, absorb, adapt to, and recover from the impacts of hazards (15, 16). Several studies have found indicators to assess disaster-resilient hospitals, including leadership, in-hospital business continuity plan (BCP), and hospital management for disaster risk reduction (17–19). Importantly, pandemic plans as part of BCP focus on enabling critical hospital functions and strengthening preparedness for a disaster (20, 21). Healthcare facilities need to prepare for and respond to crises by considering these factors.

Concerning biological hazards, hospital resilience during COVID-19 has been explored in the literature, but it is still relatively limited, including the actual participation of frontline hospitals (16, 22). There is a need for further research to enhance hospital resilience in resource-constrained and fragile settings and to offer practical guidance (16). From empirical works, a study explored hospital resilience throughout the COVID-19 recovery in the Eastern Mediterranean Region, and the need for service continuity planning was highlighted as one of the hospital roles (23). Another paper conducted in the Eastern Mediterranean Region emphasized the necessity of preventive and proactive contingency planning and strengthening preparedness capacities for such planning (24). Moreover, a recent study of hospital resilience factors in China (22) mentioned a demand for adjustment and optimization of response plans during a pandemic. However, prior studies had some drawbacks in guiding effective future actions. There was no limitation on the type or size of the healthcare facilities covered in the studies, and they did not give enough attention to the pandemic responses, considering the extent to which BCPs had been prepared in reality.

Existing research shows a wide range of aspects regarding pandemic responses, including lessons learned from experience on hospital resilience to the pandemic (24–26). An existing qualitative literature review on healthcare professionals' adaptation to COVID-19 (13), focusing on clinical settings for physical conditions, identified themes including an intensified need for individual and team capacity to handle changes in working conditions, as well as shifts in healthcare organizations to address the challenges of the pandemic. While a qualitative study focused on outbreak management by frontline workers and leaders during the pandemic in long-term care in Canada (11), many others examined psychological burdens and challenges in this setting (27, 28). To the best of our knowledge, studies considering the components of hospital resilience in vulnerable settings, especially small and medium-sized private LTCHs, during the pandemic are scarce. How these LTCHs should have navigated hospital operations rapidly in the face of limited resources during COVID-19 remains unclear. To foster LTCHs' preparedness and organizational resilience, it is important to understand how LTCHs respond to disruptions, and continue providing quality care and functioning during such unexpected crises. Addressing these research gaps, this study uses mixed methods to investigate LTCHs' perceptions of and practical responses to upholding hospital business continuity during the pandemic, considering key elements related to hospital emergency response.

## 2 Methods

### 2.1 Design

We study adopted a mixed methods approach (29) to explore LTCHs' responses considering hospital business continuity during COVID-19. This study was conducted during the COVID-19 pandemic, from August to November 2022 with cohorts of HCWs who manage the pandemic in LTCHs in South Korea. In the first phase, quantitative research was conducted via an online survey of healthcare workers (HCWs) in LTCHs in accordance with the aims of this study (Phase 1). This was followed by a second phase of qualitative data collection through semi-structured interviews with HCWs in selected hospitals to further explore in-depth views on responding to the pandemic in LTCHs (Phase 2). The researchers followed the STROBE and COREQ checklists to report this study.

### 2.2 Data collection and sampling

#### 2.2.1 Quantitative

In the quantitative phase, we conducted a cross-sectional and exploratory study. The participants in the online survey were HCWs, specifically infection control professionals (ICPs) of LTCHs. HCWs were eligible to participate if they were designated to perform infection control duties and were working in small and medium-sized LTCHs for at least the prior 6 months. Those who did not work during the pandemic were excluded. This sample considered ICPs as representatives from LTCHs, given their pivotal role in emergency preparedness, responses, and decision-making (30, 31). Using a recruitment flier, the link to the survey was distributed to LTCHs and professional associations, including the Korean Small and Medium Hospital Association (KSMHA), and the Korean Association of Infection Control Nurses (KAICN). These associations shared the link through their social networks/websites, where participants could complete the surveys. All the questionnaires were completed anonymously.

#### 2.2.2 Survey development and administration

The survey was developed with the aim of understanding LTCHs' response status to ensure continuity of operation during the pandemic. Survey questions were developed based on a review of relevant sources, including the BCP checklists provided by the Ministry of Employment and Labor (32), the World Health Organization's (WHO) hospital emergency response checklist (33), and guidelines from the Central Disaster Management Headquarters (34). The questionnaire covered domains related to business continuity planning, governance and control, communication, and resource management, including human resources and finance. For an assessment of face validity, clarity, and completeness of the survey, the questionnaire was reviewed, commented on, and revised by six experts in the field, and pilot-tested by HCWs before being implemented in the study. Following this, consensus on a final version of the survey was achieved.

#### 2.2.3 Qualitative

Semi-structured, key informant interviews were utilized to collect data. Based on an extensive literature review, an interview guide was developed to provide a general outline of the interviews and to understand the perceived and actual responses to the pandemic in LTCHs based on the findings of the quantitative phase (see Supplementary Table 1). The guide questions were devised to capture the participants' professional experiences and their hospitals' responses to the pandemic, piloted with HCWs to gather feedback on content, flow, and cohesion, and refined for contextual relevance. The guide included aspects regarding general response experience, and experiences related to disruption threats and BCP during COVID-19. For this phase, key informants were defined as HCWs, who are leaders involved in outbreak management in LTCHs; they represented HCWs in LTCHs to provide the best explanations of the pandemic responses. Purposive sampling supplemented by snowball sampling was employed to obtain a sample of HCWs, especially various team leaders with direct experience in LTCHs, including physicians, nurses, and administrators, each of with at least 6 months of experience in their respective roles. The sample size was decided considering saturation and literature (35).

### 2.3 Data analysis

#### 2.3.1 Quantitative

The statistical software package SPSS version 25 (IBM Corp., Armonk, NY, USA) was used for quantitative analysis. Descriptive statistics, including means and standard deviations, were used to summarize the responses to the questionnaires.

#### 2.3.2 Qualitative

All recorded interviews were transcribed into text, and thematic analysis was employed to analyze responses following the framework of Braun and Clark (36). The method includes six steps: familiarizing with the transcribed text; generating initial codes; collecting codes for potential themes; checking if the themes align with the coded segments; refining each theme; and writing a report with examples and quotations. Once two researchers read transcripts of all interviews, initial codes were independently created to capture recurring themes across responses. Codes were reviewed and analyzed collaboratively by three researchers (two of whom were nursing researchers; the other had methodology expertise) considering components of the WHO hospital emergency response checklist (33). Themes were reviewed and discussed by the research team. To ensure trustworthiness and validity of the findings (37), team members collaborated through several research team meetings to reach an agreement on emerging patterns, themes, and sample quotes until no new themes were identified. Coding and qualitative analyses were performed using NVivo14 (QRS International, Melbourne Australia). We used a coding framework and member checking for the credibility of this study. Peer debriefing in the research process was conducted to minimize researcher bias.

### 2.4 Ethical considerations

This study was approved by the Institutional Review Board of Kyung Hee University (No. KHSIRB-22-380).

## 3 Results

### 3.1 Quantitative findings

Table 1 summarizes the characteristics of the HCWs and affiliated LTCHs. Most LTCHs (76.2%) were small and medium-sized hospitals with fewer than 300 beds, and 95.2% had an Infection Control Department. Participants were Infection Control Professionals (ICPs) representing 21 private LTCHs. Almost all ICPs were nurses (95.2%), and more than 80% reported concurrently holding responsibilities for additional roles. The mean age of the sample was 51.00 years (SD = 8.32), and the mean years of total clinical experience was 20.46 (SD = 8.76).

**Table 1:** Hospital and respondent characteristics (*n* = 21).

**Variables**	**Categories**	***n* (%)/M ±SD**
**Hospital characteristics**		
The number of beds (median [IQR], 240 [187.50–299.50])	< 300	16 (76.2)
	≥300	5 (23.8)
Presence of an infection control department	Yes	20 (95.2)
	No	1 (4.8)
**Infection Control Professional (ICP) characteristics**		
Type of health care worker	Nurse	20 (95.2)
	Doctor	1 (4.8)
Age (range: 30–63)		51.00 ± 8.32
Total work experience (years)		20.46 ± 8.76
Time devoted to ICP role	As main role	3 (14.3)
	As part of other duties	18 (85.7)

M, mean; SD, standard deviation.

#### 3.1.1 Hospital business continuity planning

Most hospitals (85.7%) reported that they developed BCPs following government recommendations (see Table 2). Of those that had established BCPs, almost all utilized the government-provided plans by either adopting it as is or modifying it. However, 57.9% of the respondents believed that the operational status of BCPs during COVID-19 was insufficient. The reasons for difficulties in establishing/applying a BCP during the pandemic included challenges in determining the criteria for quarantine exclusion, difficulties preparing alternative staff to perform tasks, and financial issues with operating medical institutions.

**Table 2:** Business continuity planning.

	***n* (%)**
**What were the essential health services that need to be continued under all circumstances during COVID-19? (*****n*** = 25)^*^	
Inpatient wards	16 (64.0)
Outpatient centers	2 (8.0)
Hemodialysis centers	2 (8.0)
Rehabilitation services	2 (8.0)
Hospital food services	1 (4.0)
Other (e.g., isolation ward)	2 (8.0)
**Which tasks were increased or added during COVID-19? (*****n*** = 80)^*^	
Access control (e.g., managing visitors and family visits, measuring body temperature)	23 (28.8)
Related to the infection control and prevention unit (e.g., education)	16 (20.0)
COVID-19 testing	12 (15.0)
Communication with health authorities (e.g., documentation)	6 (7.5)
Making plans for managing symptomatic staff	6 (7.5)
Resources/supply management	6 (7.5)
Cleaning and disinfection	4 (5.0)
Management of COVID-19 patients	3 (3.8)
Handling complaints	2 (2.5)
COVID-19 vaccination management	1 (1.3)
Others	1 (1.3)
**What was used to establish a BCP during the COVID-19 response period?**	
The Central Accident Management Headquarters sample plan	8 (38.1)
Modification of the Central Accident Management Headquarters sample plan	8 (38.1)
BCP written by the hospital	2 (9.5)
Not formally written down	3 (14.3)
**How sufficient was the actual operational level of the BCP during the COVID-19 response period?**	
Sufficient	8 (42.1)
Insufficient	11 (57.9)
**If there were difficulties in establishing or actually applying a BCP during the pandemic, please select the reason(s). (*****n*** = 60)^*^	
Criteria for determining quarantine exceptions (quarantine period and work resumption)	14 (23.3)
Preparation and training level of alternative personnel	11 (18.3)
Difficulties operating medical institutions (finance, etc.)	9 (15.0)
Criteria for essential services and decision on reducing/discontinuing work	8 (13.3)
Lack of a cooperation system between related organizations outside the hospital	8 (13.3)
Employee agreement and acceptance of guidelines	6 (10.0)
Lack of cooperation system between related departments within the hospital	3 (5.0)
Others (e.g., no substitutes for ward shift workers)	1 (1.7)

^*^Multiple responses.

##### 3.1.1.1 Critical operations that needed to be continued

When asked about the essential functions of LTCHs, inpatient ward operations (64%), including patient admission and care, were the most frequently reported. Some respondents also mentioned outpatient services, hemodialysis centers, rehabilitation services, and hospital food services (8%, 8%, 8%, and 4%, respectively).

##### 3.1.1.2 New/intensified operations in response to COVID-19

Regarding the new/intensified operations necessary after the onset of COVID-19, access control (28.8%), including measuring body temperature and managing visitors and family visits, was the most frequently mentioned. This was followed by tasks associated with the Infection Control and Prevention Unit (20%) and COVID-19 testing (15%).

#### 3.1.2 Responses based on key components

##### 3.1.2.1 Command and control

Following the onset of the first wave of COVID-19, 71.4% of the respondents established a team responsible for infectious disease emergency preparedness and response as shown in Table 3. Only 9.5% of respondents had formed such a team before the pandemic. Regarding key decision-makers within the team, most respondents (56.3%) identified the hospital director. Over 70% reported having alternatives in the case of absent team members. Regarding operational effectiveness, 76.1% rated the team's operations as above average to excellent in fulfilling its roles.

**Table 3:** LTCHs' responses to the pandemic.

**Command and control**	***n* (%)**
**When was the infectious disease emergency preparedness (response) team formed?**	
Before COVID-19	2 (9.5)
At the onset of the first wave of COVID-19 in Korea	15 (71.4)
At the onset of the second wave	4 (19.0)
**If a rapid response was required during the COVID-19 response, who was the key decision-maker? (*****n*** = 32)^*^	
Director of hospital/head of the hospital/medical director/doctor	18 (56.3)
Director of administration	4 (12.5)
Director of nursing service	3 (9.4)
Infection control professional (infection control nurse/infection control physician, infection control committee)	6 (18.8)
Other (executive team)	1(3.1)
**Has your hospital designated alternative staff members to be delegated?**	
Yes	16 (76.2)
Yes; partially selected	1 (19.0)
Not specified	4 (4.8)
**During COVID-19, how well did the infectious disease emergency response team operate in its role?**	
Above Average to Excellent	16 (76.1)
Average	3 (14.3)
Poor to Below Average	2 (9.5)
**Communication**	***n*** **(%)/M** ±**SD**
**Please indicate how well communication and cooperation with related organizations went**	
**Degree of communication and cooperation with relevant agencies:**	
**(The higher the score, the better the overall interaction)**	**M** ±**SD**
Request for vaccine support	4.29 ± 0.21
Report on the status of infected patients	4.05 ± 0.21
Report of available beds	3.38 ± 0.24
Request for supplies and equipment	3.19 ± 0.26
Request for environmental assistance (disinfection, etc.)	3.05 ± 0.27
Patient transfer request	3.05 ± 0.25
Request for financial assistance	2.67 ± 0.27
Request for manpower support	2.48 ± 0.31
**Reasons for poor communication with relevant organizations (*****n*** = 35)^*^	
Delay in communication with related organizations	16 (45.7)
Non-unified reporting system	10 (28.6)
One-way communication	6 (17.1)
Not knowing whom to ask	3 (8.6)
**Safety: cohort isolation**^a^ **owing to the pandemic**	***n*** **(%)/M** ±**SD**
**During COVID-19, LTCHs had been put under cohort on (*****n*** = 52)^*^	
Rooms cohort	19 (36.5)
Wards cohort	19 (36.5)
Floors cohort	13 (25.0)
Entire building	1 (1.9)
Yes	19 (90.5)
No	2 (9.5)
**How much difficulty did your hospital experience during cohort isolation in the below categories?**	**M** ±**SD**^b^
Manpower management	4.33 ± 0.73
Transferring patients to hospitals dedicated to infectious disease	3.71 ± 1.06
Communication	3.52 ± 0.93
Finance	3.43 ± 0.87
Resources/supply management	3.38 ± 0.86
Leadership	2.81 ± 1.08
**Financial resources**	**n (%)**
**Has your hospital received financial compensation from the government?**	
Yes	13 (61.9)
No	7 (33.3)
Unsure	1 (4.8)
**Does your hospital have operational indicators for tracking its financial status?**	
Yes	4 (19.0)
No	17 (81.0)
**Human resources**	**n (%)**
**Ranking of staff shortage by occupation**	
**Please select the three occupations that were most understaffed during the COVID-19 response period. (*****n*** = 64)^*^	
Unlicensed care workers/assisted caregivers	22 (34.9)
Nurses	19 (30.2)
Nurse assistants	13 (20.6)
Clinical laboratory technologists	1 (1.6)
Administrative staff	1 (1.6)
Hospital cleaners	3 (4.8)
Facility maintenance staff	4 (6.3)
**Which job was the most difficult to secure skilled workers for during COVID-19?**	
Unlicensed care workers/assisted caregivers	10 (47.6)
Nurses	9 (42.9)
Nurse assistants	1 (4.8)
Hospital cleaners	1 (4.8)
**Occupation**	*n* ^*^	**LTCH reaction to the shortage (select all)** ^*^							
		**New recruitment (full-time)**	**New recruitment (part-time)**	**Dispatched personnel**	**Existing staff, no recruitment**	**Department temporary closure/ downsizing**	**Partial suspension/ reduction of services**	**Changes to operating hours**	**Outsourcing**
Unlicensed care workers/assisted caregivers	48	9 (18.8)	7 (14.6)		17 (35.4)	8 (16.7)	6 (12.5)		1 (2.1)
Nurses	37	12 (32.4)	3 (8.1)	1 (2.7)	16 (43.2)	4 (10.8)	1 (2.7)		
Nurse assistants	26	8 (30.8)	3 (11.5)		10 (38.5)	3 (11.5)	1 (3.8)	1 (3.8)	
Clinical laboratory technologists	1						1 (100.0)		
Administrative staff	2				1 (50.0)		1 (50.0)		
Hospital cleaners	8	2 (25.0)			3 (37.5)	2 (25.0)	1 (12.5)		
Facility maintenance staff	6	4 (66.7)			2 (33.3)				

^a^Cohort isolation is a method for containing the COVID-19 by putting health care workers and patients under group quarantine.

^b^The extent of difficulty respondents experienced was measured on a 5-point Likert scale, where higher scores indicates that they experienced a greater degree of difficulty.

^*^Multiple responses.

##### 3.1.2.2 Communication

Between LTCHs and relevant agencies during COVID-19, the most effective communication was related to requests for vaccine support (4.29 ± 0.21), while the least was related to requests for manpower support (2.48 ± 0.31) and financial assistance (2.67 ± 0.27). Reasons cited for poor communication with relevant agencies (such as public health centers and local governments) included communication delays, non-unified reporting systems, and one-way communication.

##### 3.1.2.3 Cohort isolation

All respondents reported that their hospitals implemented some form of cohort isolation (including types of isolation at the room and ward levels, floor level, and the entire building) during COVID-19. Although a substantial number of LTCHs (90.5%) had prepared manuals for implementing cohort isolation, they encountered several challenges. The most difficult one was managing manpower (4.33 ± 0.73), followed by transferring COVID-19 patients to hospitals dedicated to managing infectious diseases (3.71 ± 1.06).

##### 3.1.2.4 Human resources

Considering the rankings individually, staff shortage was most severe among nurses, then among unlicensed hospital-based care workers (ganbyeongin)/care assistants, and then among nursing assistants. Furthermore, when combining the shortfalls in staffing for the three highest ranks, LTCHs experienced staff shortages, with unlicensed care workers (ganbyeongin)/care assistants (34.9%) being the most frequently reported, followed by nurses (30.3%) and nurse assistants (21.2%). Additionally, the jobs that were most difficult to find skilled individuals for during COVID-19 were nurses and unlicensed care workers/care assistants. In response to staff shortages, most LTCHs explored various options, including operating with existing staff without recruitment, new recruits, and partial the suspension/reduction of services.

##### 3.1.2.5 Financial resource

More than half of the LTCHs (61.9%) reported receiving financial compensation from the government to mitigate losses incurred due to COVID-19. Nevertheless, the majority (81%) did not monitor indicators that reflected their financial status.

### 3.2 Qualitative findings

Eleven semi-structured interviews were conducted. The participants were frontline leaders and middle managers responsible for leading staff, such as hospital directors, directors of nursing and food services, and other health care workers (e.g., nutritionists, physical therapists, and administrative staff). The mean age of the respondents in our qualitative sample was 48.91 years (SD = 9.35, range = 36–66), and the mean years of clinical experience was 16.64 years (SD = 10.88, range = 2–42); most were female (63%, *n* = 7). The participants' information and summary of responses to identified themes are presented in Supplementary Tables 2, 3.

Six main themes, with each theme having sub-themes were identified from the interviews regarding responses to COVID-19 to maintain LTCHs' operational continuity. The six themes were: workplace culture and leadership, communication, human resource, safety, continuity of essential service, and financial and supply resources. The following interpretations demonstrate the themes along with participants' statements.

#### 3.2.1 Theme 1: workplace culture and leadership response

In LTCHs, workplace culture and leadership were considered forces alleviating the challenges of the pandemic in settings with scarce resources to plan and prepare. The efforts of the hospital staff members and the strong leadership structure centered around the hospital owner and director in small and medium-sized hospitals were related to this response.

##### 3.2.1.1 Responding to the crisis based on the strong bond between staff members

Workplace culture response was manifest through strong cohesion and a culture of collective orientation, a mutual cooperative atmosphere, and employees' sense of responsibility. LTCHs in which pre-pandemic workplace culture was positive continued to benefit from these influences during the pandemic. This atmosphere helped the staff endure the circumstances by inspiring a sense of community and voluntary sacrifice. The following is an example:

“All the staff members had a sympathetic heart. They worked long hours and came to work to cover each other's duties. Even the director of nursing hadn't been home for almost two weeks and continued to take care of patients on the ward. Before the Omicron variant, the hospital was completely locked down and nobody could go home. It was not because they caught the virus, but because there were many people who volunteered to stay and do their work” (Director of nutrition services).

##### 3.2.1.2 Dealing with the pandemic through hospital leadership

Crisis leadership was identified as an indispensable strategy to prevent the disruption of privately-owned LTCHs. As an important leadership response, hospital leaders' behavior described by participants included their high levels of consideration, ability to instill trust among their members, and attempts to be a good role model. Leaders with these attributes showing how to respond to crisis by example can become a source of endurance and a facilitator for their intense work together. One stated that:

“In any case, for the shifts to be covered, there needs to be a minimum staffing level... So, for those gaps, the nursing supervisor or doctors can just... I, as the hospital director, have had to come and go a bit at night without sleeping. As a director, I thought, ‘I really have to show that I'm willing to go through hell. I have to be the first to endure it.' Otherwise, it wouldn't work. So, it's literally like that. If the captain of a sinking ship is the first to run away, then everything just falls apart, right?” (Hospital director).

#### 3.2.2 Theme 2: communication response

Multiple HCWs expressed how they responded to the situation by communicating with relevant stakeholders and neighboring LTCHs for decision-making and collaboration. They mobilized the communication channels they had been using in their personal sphere for their institutional response during the pandemic.

##### 3.2.2.1 Expanding intra-organizational communication via social networking platforms

As 24-hour communication channels, mobile technology and social network services (SNS) helped provide better communication between HCWs in LTCHs. Participants stated that while larger tertiary hospitals tended to have their own internal intranet and communication channels, smaller providers relied on freely available SNS, just like they did. Participants also remarked how they communicated with each other using SNS/mobile apps to respond quickly, and share information with various departments inside the organization, including head nurses, administrative departments, and team leaders. This was reflected in responses such as, “Announcements about constantly changing responses... We use KakaoTalk.” (Director of Nursing) This facilitated communication, in turn, became possible to support LTCHs' communication with health authorities, including public health centers.

##### 3.2.2.2 Expanding inter-organizational communication via social capital

Information about the pandemic, including practice guidelines for treatment and management that reflect real situations, was limited and not readily available. It was crucial to obtain and share the know-how for HCWs to respond to this newly emerging virus and its impact. Participants explained this interaction:

“For those with more severe conditions, I have heard Remdesivir works better. Since we began exchanging information with other LTCH directors, we also tailored treatments with dexamethasone when necessary. We do not just follow the guidelines from the Disease Control and Prevention Agency; our approach is grounded in our real, firsthand experiences. By acting on the collective experiences of directors, we have seen a significant reduction in patient severity” (Hospital director).

#### 3.2.3 Theme 3: human resource response

The third theme, human resource response, captures a situation in which a large number of care staff is required in LTCHs due to the high number of long-term bedridden patients. Human resource responses were the most frequently reported. This theme encompassed utilizing short-term recruitment strategies for prompt response, division of duties among existing employees, efforts to retain staff through compensation and support, and multifaceted efforts to minimize employee exposure to infection risk.

##### 3.2.3.1 Recruitment: utilizing short-term recruitment strategies for prompt response measures

Due to LTCHs' difficult working conditions and low wages, recruitment was cited as an ongoing challenge that existed before the pandemic, but worsened during the pandemic. In particular, for unlicensed care workers, LTCHs preferred to hire ethnic migrant workers mostly from China (Joseonjok) and Central Asia (Koryo Saram), because domestic care workers were hard to find and had higher wages even before the pandemic. Restrictions on traveling due to COVID-19 prevented them from returning to Korea, further exacerbating staffing levels. To address staffing issues, stopgap measures were taken, including hiring unlicensed care workers (ganbyeongin) by increasing daily wages, replacing nurses with nurse assistants, and checking the availability of temporary staff with contracts at other hospitals or with the government. Some quoted issues related to care workers and nurses.

“The director just called in external care workers (ganbyeongin) who had either tested positive for COVID-19 or had cared for COVID-19 patients. If they were paid around 100,000 won a day at other hospitals, we paid them 300,000 won, even if it meant spending more money. So, we tried to keep them for a few days. But these people don't have any attachment to our hospital.” (Director of nursing).

“Many RNs quit…so, I said that I would manage the hospital with the nurse staffing grade 1 (based on nursing staff-to-patient ratio) without nurses, only with our nursing assistants.” ^*^Nursing staffing grades go from 1 to 7, with 1 being the highest nurse staffing level (as an indicator of the quality care) (Director of nursing).

##### 3.2.3.2 Workforce arrangement: division of duties among existing employees

Deployment of existing health workers by changing the arrangement of staff and their duties was required due to the shortage of available HCWs. Division of duties among current staff included multitasking, modification of employee quarantine period depending on the situation, operation centered on middle managers (e.g., head nurses), patience of non-infected staff members until the quarantine period of the COVID-infected staff ends, working with adjusted schedules (e.g., double shifts, overtime, reduced break time, and solo night shift), and the handling additional tasks related to infection control. Many respondents described additional roles beyond their existing responsibilities (e.g., multitasking) in the care environment.

“Physical therapists supported others. Like... (in a soft voice) packing lunchboxes or something along those lines. For me, it was my first time dealing with COVID-19, and honestly, transporting COVID patients... We didn't have to usually do the disinfection in that way, because there is a company for that. But, because of COVID-19, we had to do all the disinfection ourselves” (Administrative staff).

“Actually, it was tough, but during that time, we had to work without enough staff, so all the staff ended up doing double shifts, and head nurses were deployed for night duties, and so on” (Infection control nurse).

##### 3.2.3.3 Workforce retention: efforts to retain staff through compensation and support

It is vital to retain staff for operating hospitals and providing quality care. During the pandemic, compensation and support were frequently mentioned strategies, as the pandemic amplified HCWs' stress and turnover. Participants reported financial compensation as a motivational measure, as well as efforts to support and comfort employees (e.g., buying snacks or supporting staff by verbally motivating them).

“While there weren't salary increases or anything like that, we did provide a lot of snacks and engaged in a lot of conversations with the intention of saying we were all in this together, trying to overcome it collectively. Additionally, we have team leaders for care workers (ganbyeongin) now. We asked these team leaders to provide some extra care for them” (Administrative staff).

##### 3.2.3.4 Minimization of employee exposure to infection risk

LTCHs took a multifaceted approach to prevent staff shortages due to staff infections and their isolation. These included encouraging more HCWs to receive COVID-19 vaccination, performing extra self-testing to avoid being the source of infection, minimizing non-essential interactions among current staff, limiting excursions to crowded places during time off, supplying PPE, and educating staff. Through these measures, they not only protected the workforce, but also ensured the continuity of hospital operations.

“Um... somewhat, (nervous laugh) in a slightly coercive manner, like taking pictures of each employee at around 8 or 9 in the evening to ensure they were at home. We implemented a bit of control, not exactly control, but more like monitoring, I guess” (Administrative staff).

“Other departments may follow guidelines, conducting PCR tests twice a week at the public health centers and self-testing with antigen kits three times a week. In our department, in addition to those guidelines, we perform self-testing with antigen kits every day before coming to work …Furthermore, we monitored temperature changes in the morning, afternoon, and evening” (Director of rehabilitation).

#### 3.2.4 Theme 4: safety response

To reduce the number of infected patients and fatalities caused by COVID-19, safety measures were noted to be critical responses. These measures include changes in decisions regarding transfers and infection prevention for patients and visitors.

##### 3.2.4.1 Changes in decisions regarding transfers

During the early stages, the rapid transfer of infected patients to designated hospitals was considered the first approach for controlling infection and caring for infected patients. Later, as transfer delays occurred due to the capacity limitations of designated hospitals, the participants reported associated concerns and changes in responses.

“At the very beginning, when beds were still available at the designated hospitals, they would admit the patients promptly. However, as time passed, the rooms for transferring COVID-19 patients did not become available. There was a time when we had to wait for up to three days. Initially, when it happened, we managed like that, but later on, the transfers were too delayed...Even when they were transferred to the designated hospitals, they (the designated hospitals) did not look after the patients as...(pauses) well as we did. They do not care as well; therefore, we decided to take care of them ourselves and provide proper treatment” (Infection control nurse).

##### 3.2.4.2 Infection prevention for patients and visitors

Proactive activities related to preventing infection used by LCTHs involved organizing isolation areas, disinfecting environments, ventilation by opening windows, vaccinating patients, conducting mandatory preemptive PCR testing/comprehensive patient testing, strictly controlling outsider access (vendors, visitors). Amid the changing situation and evolving visitation guidelines announced by the government, the implementation of family visits was particularly important in LTCHs. Adjusting the visitation methods (e.g., contact-free visits and video calls) to fit the situation was necessary. For instance, one shared:

“We conduct (non-contact visits) every time. The patient's family makes a reservation in advance and brings a rapid test kit. (After conducting the COVID-19 rapid test), they then wear protective clothing like a PPE gown and gloves, and change their masks, before proceeding downstairs like that. (The family) meets the patient from outside the transparent barrier... For setting up non-contact visits, we tried everything…It took several tries to get it right” (Infection control nurse).

LTCHs had to address the vulnerability of physical spaces within LTCHs to accommodate the increase in infected patients and the remaining uninfected patients. HCWs described difficulties in securing designated pathways to separate clean and contaminated areas. One of the efforts to use a separate entrance/route was to avoid using the elevator; instead, stairs were used when transporting supplies.

“Since there was only one elevator, we had to bring down the waste. All the meals were provided to the ward in disposable containers, but the thought of putting those containers in the elevator made us feel like something might come out of it. Really. Even after disinfecting, we were still scared, so we carried everything up to the 4th floor using the stairs, every single meal. All the staff... We kept doing it throughout the entire cohort isolation period...” (Infection control nurse).

#### 3.2.5 Theme 5: response to the continuity of essential services

Participants experienced the discontinuation of unnecessary functions and a lag in the development of the action plan for maintaining essential services.

##### 3.2.5.1 Suspension of non-essential functions

Identifying priority functions in the context of the pandemic led to the closure of non-essential services. In addition to medical care, some respondents considered hospital food service operations, medical billing claim reviews, and personal care from non-licensed care workers (live-in care workers providing ongoing care) difficult to replace. HCWs described how prioritization could be applied.

“Everyone was still coming to work, but we had to close the rehabilitation room. That was the only place we could close. Patients from the second and third floors got mixed up [in the rehabilitation room]. Oh, there is one more place. Traditional Korean medicine treatment is also considered part of the supplementary services, you know...That is why we had to stop operations at those two places” (Director of nursing).

##### 3.2.5.2 Shifting perceptions of response planning

Regarding the activation of hospital responses, efforts to devise operational plans assuming the impact of the COVID-19 were noted. Ad hoc planning after the occurrence of the infected patients was cited. HCWs experienced increased awareness of the severity and the need for action planning after the emergence of their own confirmed cases.

“From the time we got our first patient, we started making more specific plans. Before that, we had a bit of an attitude of 'it won't happen to us.' We hadn't had any cases for a long time, so we trusted that everyone was doing well and believed we wouldn't be affected. But after we had a case, we started making two-week plans continuously from that point on” (Administration staff).

#### 3.2.6 Theme 6: response to financial and supply resources

Handling the loss of financial resources and maintaining essential supplies were important issues for the emergency response during the pandemic.

##### 3.2.6.1 Bearing losses with constrained finances

LTCHs experienced a decline in patient admissions, which led to a decrease in revenue. The decline in patient admissions was due to the stigma of being seen as a hospital connected to the outbreak and to patients not returning after being transferred to designated hospitals. Importantly, insufficient contingency funding, which did not account for potential financial impacts/barriers in planning seemed to have had an unfavorable effect on the response capacity.

“The chaos caused by COVID-19 is almost unprecedented. This was the case across the country, and hospitals probably approached it with a mindset of accepting some level of loss and expense. [We] likely aimed to minimize this as much as possible. The reserve funds were only held to the extent required for achieving LTCHs accreditation. Since COVID-19 was rapidly spreading, we were in a position where we had to prevent it. As a result, we had to act quickly, which inevitably incurred costs” (Director of administration).

The government required accurate documentation and evidence of LTCHs' response activities, including waste disposal costs, which led to delays in receiving compensation or resulted in some LTCHs being unable to receive compensation or receiving only partial compensation. One described difficulty in proving their losses for reimbursement.

“We were only able to repay half (of the loan) due to the actual loss. The money we claimed from the public health center and the Health Insurance Review and Assessment Service—well, for waste disposal and so on—is difficult to prove… It was difficult to compare with the waste from before” (Infection control nurse).

##### 3.2.6.2 Efforts to secure uninterrupted supplies

Some protective equipment was partially provided by the government. However, because it only served as a supplement, informants expended considerable effort on supplies by searching for available sources, such as via the Internet and public organizations, and spending a lot of money to maintain supply levels to manage situations. It was related to the lack of preparedness in identifying and securing suppliers for essential medical supplies.

“…Since the supplies were tightly managed, we also ordered a lot of motorcycle couriers (called quick service). When regular delivery was not possible, we paid extra and used lots of same-day delivery. Things like face shields are indispensable. Initially, we discarded them after use, but later, we wiped and reused them” (Infection control nurse).

### 3.3 Integration of quantitative and qualitative findings

The integration of qualitative and quantitative data provided greater insights into LTCHs' responses for the continuity of healthcare services. The findings from the qualitative phase, providing contextual meaning and examples, confirmed and expanded the quantitative findings. Table 4 presents a joint display of the pandemic responses among LTCHs based on the integrated analysis.

**Table 4:** Joint display of quantitative and qualitative results.

	**Summary of quantitative results**	**Summary of qualitative results**	**Mixed methods-inferences**
Hospital business continuity planning	(BCP) The most frequently mentioned critical operation was inpatient care, while one of the least mentioned choices was rehabilitation service. (BCP) Almost all (85.7%) developed BCPs according to government guidelines, but 57.9% of them felt the operational status was insufficient.	(Theme 5) Suspension of non-essential functions: LTCHs tended to close rehabilitation services and traditional Korean medicine treatment, which were considered nonessential services and potentially sources of infection. (Theme 5) Shifting perceptions of response planning: LTCHs tended to underestimate the risks and, after having their own COVID-19 cases, started making specific plans.	Confirmed/Expanded: LTCHs identified inpatient ward operations that needed to be available under any circumstances. The qualitative phase explained how the services to be postponed were selected, reflecting the need for ranking services considering priority in BCP. The qualitative data revealed the underlying reasons for their BCP operational status. Amid the ongoing pandemic, the government asked LTCHs to create a plan, so there was not enough time to review or prepare it.
Command and control	(Command and control) Most response teams (71.4%) were formed after the first outbreak. The hospital director was considered the primary decision-maker in more than half of the cases. The majority of teams (76.1%) scored above average for operational effectiveness.	(Theme 1) Responding to the crisis based on the strong bond between staff members: A positive work climate fostered a sense of camaraderie that laid the foundation for better operation. Dealing with the pandemic through hospital leadership: being dedicated and leading with consideration as a leader was a helpful strategy for responses.	Expanded: the qualitative data might add valuable insights into reasons why the command group did not score lower for operational effectiveness and how LTCHs responded through leadership and workplace culture, even though the team was not set up ahead of the outbreak.
Communication	(Communication) Communication difficulties with external agencies were due to delays in communication, lack of integrated reporting systems, and top-down communication.	(Theme 2) Expanding intra-organizational communication via social networking platforms, which enabled timely communication among staff. Expanding inter-organizational communication via social capital: Exchanging their experience with other LTCHs had a positive effect on informed decision-making.	Expanded: In addition to communication barriers with public health agencies arising from quantitative data, both themes capture how LTCHs communicated with other stakeholders. The qualitative data, whereby HCWs described not only intra-organizational communication but also communication between other LTCHs, exemplify efforts that facilitated communication and collaboration through the use of informal resources.
Safety	(Cohort isolation) All participants, representing each LTCH, reported experiencing cohort isolation in one form or another. Transferring patients to dedicated hospitals, was identified as one of the causes of difficulties. (BCP) The most frequently reported new/intensified operation was related to access control for safety, especially managing visitors.	(Theme 4) Changes in decisions regarding transfers: Delays in sending infected patients and the deterioration of care quality in the designated hospitals forced LTCHs to accept infected patients. Infection prevention for patients and visitors: Because the spaces were not originally designed with the pandemic in mind, HCWs endeavored to implement containment strategies, including contactless visits and alternative routes, to prevent the spread of contamination.	Confirmed: The qualitative findings illuminated how LTCHs responded to transport delays and spatial vulnerability to ensure hospital safety and maintain functionality.
Resources	(Human resources) The occupations with the most severe shortages and the greatest difficult in finding skilled workers were unlicensed care workers and nurses. Measures taken to address the staff shortages were identified. (Communication) The question asked how well communication with related organizations received the lowest score in the request for manpower support category. (Cohort) High levels of difficulty in manpower management were identified during cohort isolation.	(Theme 3) Utilizing short-term recruitment strategies: Pre-COVID recruitment difficulties, including reliance on the employment of migrant workers, led to a focus on short-term solutions. Division of duties among existing employees: Overworking existing staff beyond their roles Efforts to retain staff through compensation and support in an attempt to boost motivation. Minimization of employee exposure to infection risk: giving extra attention, including additional self-tests and restrictions on outings, was necessary to prevent staff infections, avoiding the worsening of staffing shortages.	Confirmed: Qualitative data provided context and explanation for LTCHs' shortage of unlicensed care workers and nurses identified in the survey. Qualitative work indicates that LTCHs with high caregiving demands faced staffing issues due to the lack of a systemic staff management and supervision system. Reactive self-help measures, rather than fundamental solutions, were implemented to mitigate the prevalent staffing challenges, supported by qualitative data. These may have come at the cost of an increased burden on existing staff and a decline in the quality of care.
Resources	(Financial resource) More than 80% of LTCHs did not have indicators to monitor their financial status. Approximately 62% reported receiving financial compensation from the government.	(Theme 6) Bearing losses with constrained finances: With limited funds caused by insufficient planning, LTCHs aimed to minimize losses and expenses. Compensation from the government was limited by the need to provide evidence of the loss incurred in the course of the response activities, leading to insufficient compensation.	Confirmed. Qualitative data confirmed these findings and provided details on the situation in which tracking financial indicators and potential compensation were not preemptively considered. The lack of proactive estimation and planning for contingency response funding contributed to a cost-savings approach. The rigid government compensation system may have complicated the responses.
Resources	(BCP) One of the reported new/intensified operations concerned resource and supply management.	(Theme 6) Efforts to secure uninterrupted supplies: The government's support was helpful, but LTCHs invested considerable time and money in maintaining essential supply levels.	Expanded. Qualitative data showed that LTCHs were forced to rely on unstable supply chains in relation to the lack of an existing supply management plan, including lists for suppliers (e.g., key, secondary, and backup).

## 4 Discussion

This study shows a range of pandemic responses among HCWs in LTCHs from a mixed perspective, considering both quantitative and qualitative data. This study applied qualitative data to gain more detailed information about responses in order to explain and complement the quantitative findings. This study can enhance our understanding of their circumstances and assess whether any components were left out in the responses.

Regarding BCP and response to the continuity of essential service, both quantitative and qualitative data indicate that in LTCHs, inpatient services were perceived as a critical function. However, they experienced some disruptions to rehabilitation services, which were deprioritized compared to other primary care services during the pandemic. Rehabilitation rooms were at risk of spreading the infection due to the need for direct patient contact and the high possibility of patients mixing together. The grounds for selecting operations in LTCHs were supported by qualitative data, which provided additional insights. Different from LTCHs in Korea, hospitals in Delhi (18) identified the emergency department as a critical function, as it is based on hospital type and specific circumstances. During the pandemic, a recent study showed that a hospital in Singapore identified orthopedic surgery as an essential service, reviewed and postponed non-urgent elective surgeries, and reduced outpatient visits to continue providing core musculoskeletal care (38). Further to this, during the pandemic, despite local differences in the choice of which specific services to maintain, hospitals prioritized essential services and canceled/postponed non-urgent care in countries in the WHO European region and Canada (39). In health service continuity planning as an ongoing and proactive process, identifying critical functions and recovery time objectives for each hospital based on its unique situation is necessary, and thus helps LTCHs reprioritize resources and efforts to continue their essential services (19, 40).

In addition, we found that many LTCHs quickly created BCPs using sample plans, but their operational status appeared to be unsatisfactory. The qualitative results, in which HCWs described their experiences of making action plans once they received their first confirmed case, help explain inefficient BCP implementation. The combined data showed that they may have experienced difficulties in building and implementing BCPs in situations with limited time and resources, including setting criteria for quarantine and essential services. While developing and testing BCPs tends to take several months, LTCHs in Korea had to create BCPs within just a few weeks for the first time because the government urged planning due to the pandemic. This has been criticized for mainly focusing on the reduction of isolation periods rather than properly reflecting the realities in each case (41, 42). Similarly, during the response to COVID-19, as their initial response, many countries focused on planning in hospitals; some countries determined the overall response, while others provided guidance, and left individual hospitals responsible for how they implemented the measures (39). Therefore, LTCHs should plan for a potential pandemic with a permanent BCP committee, taking the time to develop a BCP that covers all the critical components and periodically updating it (19, 20); in turn, their BCP would be effectively implemented during actual crises.

The WHO (33) emphasizes that well-functioning command-and-control systems are related to effective emergency management operations. In the quantitative data, LTCHs reported that response teams were mostly formed after the COVID-19 outbreak, but the operational effectiveness of most teams did not fall below average. Qualitative data provided further explanation of the response team's operation through workplace culture and leadership. Many HCWs reported that doctors who are owners/founders of hospitals were called directors/heads of the hospital. Due to this decision-making structure, implementation in an outbreak response can be made quickly without the approval of other parties. Global studies in the COVID-19 era corroborate that, because crisis management depends on hospital leadership, leaders should be trained to improve their inadequate leadership skills and serve as a champion (23, 43, 44). Consistent with our findings, a previous study (24) found that strong leadership with a clear vision can help push a hospital through the pandemic; the leadership of middle management also needs to be equipped to improve hospital outbreak response, since middle management is responsible for executing much of the day-day work (24). Moreover, during the pandemic, respectful organizational culture focusing on team-based approaches also fostered resilience for outbreak response, which is consistent with a previous study in long-term care (11). This implies that workplace culture and organizational leadership may hold a significant portion of the key continuity strategies and resources of private LTCHs.

In terms of communication, quantitative data showed that LTCHs reported a lack of smooth and effective risk communication between LTCHs and local health authorities. Meanwhile, qualitative data indicated that intra-hospital communication was relatively smooth when using smartphone technology, and sharing information with other LTCHs was beneficial for pandemic responses. The combined data highlighted a need to establish and activate a system with clear communication channels between authorities/stakeholders and LTCHs for real-time access, information exchange, and updates without delay or confusion. Some of these results echo the findings of a study that, through a multinational survey, pointed to increased use of virtual technologies for communication among health professionals, in parallel with changes during the pandemic, to keep staff connected and up to date with the ever-changing situation (45). Therefore, it is important to include information on communicating with stakeholders in BCP and provide support to address obstacles in order to ensure effective communication across and beyond hospitals during crises (40, 46).

Concerning the safety response, of the objectively measured new/intensified tasks, access control for safety accounted for the highest proportion. LTCHs reported implementing cohort isolation and experienced a high degree of difficulty in transferring patients during the process. The qualitative data confirmed these findings and revealed vulnerabilities and a process of trial and error in LTCHs. LTCHs in Korea lacked well-equipped isolation rooms and infection control specialists, so they had no choice but to follow the policy of transferring confirmed patients to hospitals dedicated to infectious diseases in the early phases, despite concerns over the quality of care in those hospitals (47). Later, a small number of designated hospitals with limited capacity were unable to admit confirmed patients, which led to transfer delays and the possibility of a surge in cluster infections in LTCHs (48). This is consistent with a study that found that several countries designated specific hospitals for the receiving and treatment of COVID-19 patients, but as case numbers increased, this arrangement was adapted, requiring other hospitals to admit confirmed cases as well (39). In addition to the lack of negative pressure isolation rooms, the lack of infection prevention and control (IPC) facilities, including single occupancy rooms for isolation, clear route separation, mandatory installation of ventilation systems, and a novel type of separate visiting area, further complicated the response to safety concerns (9, 49, 50). Taking this situation seriously, the government should provide financial and policy support to ensure the installation of IPC-related facilities, the activation of non-contact visits, and the proper layout of spaces in LTCHs, in preparation for future pandemics.

This study shows the pandemic placed a substantial burden on resource responses, pushing LTCHs to their limits, mirrored in other studies (11, 13, 20, 51). In particular, manpower issues throughout the pandemic response were frequently identified as the biggest challenge. The qualitative results in which HCWs described their experience of addressing human resources, largely confirmed and provided details on various self-help solutions. From this combined data, inferences emerged that pointed to desperate efforts to prevent staff shortages due to staff infections, as well as the impact of the pre-existing staffing issues of small and medium-sized LTCHs in Korea, including the absolute shortage of HCWs and instability in workforce quality, resulting in reduced care quality. Research involving hospitals in five countries (25) demonstrated comparable results; in terms of human resource management, during the pandemic, recruitment and retainment strategies were emphasized, including task-shifting with administrative staff assisting other services, recruitment of short-term contractors, moving staff within facilities, and simplifying recruitment, leading to hiring poorly qualified staff and less effective reinforcement in subsequent waves. This stresses the necessity for establishing a staff management and supervision system through collaboration between LTCHs and health authorities, including a human resources roster for infectious disease control and treatment, rather than counting on short-term solutions from individual LTCHs (25, 33). Considering the issues with care policies in Korea, improving the care worker system is also required, including the coverage of care worker costs through the National Health Insurance (48). Moreover, rewards for staff described during interviews, supporting and protecting staff can act as an important buffer against higher workload. Along with band-aid solutions for meeting immediate needs, better responses could be achieved through medium- to long-term strategies and planning, including needs assessment, ensuring an appropriate workload, and the development of incentive packages (52).

Quantitative results identified that in LTCHs, monitoring financial indicators was lacking in practice, and receiving compensation from the government was also difficult. Qualitative data provided more details insights into their limited financial responses. This meant that fiscally strained LTCHs, without a financial plan and plan evaluation, would not be able to provide adequate care to both infected patients and the remaining patients during the pandemic. The literature in this area stressed the need for increased fiscal autonomy for effective responses during the pandemic (24). Identifying funding sources as well as planning flexible contingency funds and reimbursement beforehand are foundations to meet additional demands for response (40). Also, additional financial compensation for hospitals' financial losses was a motive in other countries (53), so the government should develop payment systems to implement compensation for pandemic-related losses quickly, directly, and with great attention to distressed LTCHs.

How supply responses were made to address the continuity of the LTCHs' supply was provided by qualitative data. This may be related to the fact that the need of supplies was recognized without sufficient time to estimate or plan it in advance, consistent with previous research (47). Inconsistent assistance for PPE from the government and limited finances may also have an impact on supply management in LTCHs during the pandemic (28). This result aligns with earlier studies showing that tensions related to uncertainty about supplies were experienced due to a lack of diversity of supply channels, the absence of an inventory management tool, and shortages in the market, resulting in delivery delays and efforts to minimize unnecessary consumption and material waste, as well as efforts toward needs-based distribution (13, 24, 25). In this regard, evidence on hospital resilience has pointed to the importance of the government's proactive preparedness and comprehensive contingency planning, including the acquisition of supplies (23). Based on this lesson, it is important to plan for supply vulnerabilities at both the facility and national levels, including establishing a centralized supply system for the LTCHs through the health authority and alternative supply chains, maintaining reserves, and identifying storage space, to mitigate shortages of supplies (20).

Novel perspectives on LTCHs' BCP and responses to the pandemic in practice were gathered from HCWs, which contributes insights for developing and guiding preparedness plans and policies for vulnerable healthcare facilities in similar contexts, not only in Korea but also other countries, and provides evidence to enhance future responses. Similarly, studies suggest that small healthcare facilities should have BCPs to mitigate the impact of disasters, as damages (including preventable disaster death) occur in most hospitals with fewer beds, and patients cannot differentiate between the services available (18, 19, 54). It is thus imperative to be well prepared, including adaptive leadership, resilient staff, and established contingency funds for sustainable financing, regardless of hospital size (23, 55).

Nonetheless, its limitations should be acknowledged. First, our sample size was relatively small albeit focused on HCW representatives from LTCHs, restricting the conduct of additional inferential analyses. The low number of evaluated LTCHs in Korea also limits the generalizability of the results and findings; however, this indicates that this is an exploratory study, and further research is needed to gain a clearer understanding of responses in specific aspects and relevant factors in LTCHs. Moreover, this initial and exploratory study provides important information on this topic, but our study could be affected by the potential for selection bias. To obtain a representative sample for quantitative data, we attempted to recruit HCWs through professional associations/platforms used by small and medium-sized hospitals; however, recruitment was challenging during the pandemic, and self-selection bias cannot be prevented. While the selected small and medium-sized LTCHs are typical response cases in Korea, the interview participants may not represent the entirety of LTCHs. These may impact the generalizability and credibility of the findings. Future studies with more rigorous sampling methods, such as stratified random sampling, could be used to obtain more representative samples. Another limitation is related to the timing of data collection, which can lead to recall bias. In light of the persistence of the pandemic up to the Omicron period, HCWs were asked about their overall experiences while responding to the pandemic a few years after the initial outbreak. Additionally, a one-time survey of HCWs is limited in specifically capturing recovery phases and the changes across multiple waves of the COVID-19 pandemic, not the overall responses.

## 5 Conclusion

This study provides an opportunity to capture lessons learned by LTCHs that did not internalize BCPs when responding to crises. To protect vulnerable patients, ensuring the continuation of the LTCH operation is vital during a pandemic. The key lesson is that planning that fits the needs of the LTCHs and BCP implementation are critical to the LTCH's response and ultimately resilience. Considering BCP elements of planning within LTCHs is necessary to identify strong and weak points to prepare for and respond to future pandemics. To improve organizational resilience in LTCHs, there is a need for comprehensive continuity management strategies, including innovative platforms and policies, to support devising and implementing a realistic and effective contingency plan, and ensuring ongoing preparedness. At the same time, based on the findings, LTCHs need further adjustments and enhancements in their BCPs along with live, structured action plans, and should embed BCPs into the organization's culture.

## Data Availability

The data supporting the findings of this study will be made available by the corresponding author upon request.
