# Supplementary material for: Healthcare workers' views on the response to COVID-19 in long-term care hospitals in Korea: a mixed-method study
Source: Front Public Health. 2025 Jun 16;13:1518998. doi: 10.3389/fpubh.2025.1518998 (PMC12206829; doi:10.3389/fpubh.2025.1518998)
Supplement: Supplementary file 1 [file Table_1.docx]

**Supplementary Table 1.** Semi-structured interview guide

| **Semi-structured questions** |
| --- |
| **Opening question**  Could you please share the most significant experience you had while working during the COVID-19 pandemic?  **Key questions**  Could you please share your overall experience with how your hospital responded to the COVID-19 pandemic?  In relation to your hospital's response to the COVID-19 pandemic, which aspect do you think was handled the best? Why do you think so?  What do you think was the most challenging aspect of your hospital's response to the COVID-19 pandemic? Why do you think so?  Could you please share your experiences of feeling a threat to the continuity of hospital operations during the COVID-19 pandemic?  Could you please share your experiences related to managing personnel during the COVID-19 pandemic?   - How did you manage to secure staff? - How did you cope when you could not secure enough staff?   Could you please share your experiences of handling a confirmed patient during the COVID-19 pandemic?  Could you please share your experiences of managing a situation where a staff member tested positive for COVID-19 during the pandemic?  Could you please share your experiences of following government guidelines during the COVID-19 pandemic?   - Prohibition of visitation/implementation of non-contact visits - Cohort isolation - Other guidelines   Could you please share your experience related to the Business Continuity Plan (BCP)?  **Closing question**  Is there anything else you would like to add? |

**Supplementary Table 2**. Profile of interview participants

| Code | Gender | Age | Position | Career, year |
| --- | --- | --- | --- | --- |
| A1 | Male | 45 | Physician | 21 |
| A2 | Female | 46 | Nurse | 22 |
| A3 | Female | 52 | Nutritionist | 15 |
| A4 | Male | 45 | Administrative staff | 10 |
| A5 | Female | 36 | Physical Therapist | 15 |
| S6 | Female | 66 | Nurse | 42 |
| J7 | Female | 63 | Nurse | 15 |
| J8 | Female | 50 | Nurse | 10 |
| J9 | Male | 49 | Physician | 25 |
| J10 | Female | 50 | Nutritionist | 2 |
| J11 | Male | 36 | Administrative staff | 6 |

**Supplementary Table 3.** Summary of responses to identified themes

| **Theme** | **Sub-theme** | **Codes** |
| --- | --- | --- |
| Workplace culture and leadership | Responding to the crisis based on the strong bond between staff members | Strong cohesions and a culture of collective orientation, mutual cooperative atmosphere, employees’ sense of responsibility |
|  | Dealing with the pandemic through hospital leadership | The hospital leaders’ behavior with a high level of consideration, members’ trust in the leader, leaders strive to be a role model |
| Communication | Expanding intra-organizational communication via social networking platforms (inside the organization) | The use of SNS (e.g., LINE, KakaoTalk) and mobile app (e.g., SeeGene app for COVID test results) to facilitate communication and connection among staff |
|  | Expanding inter-organizational communication via social capital (outside the organization) | The utilization of hospital networks to gain know-how and advice from colleagues with direct experience |
| Human resource | Recruitment: Utilizing short-term recruitment strategies for prompt response measures | Checking the availability of temporary staff with contacts at other hospitals/with the government, replacing nurses with nurse assistants, hiring unlicensed care workers by increasing daily wages, preference for experienced candidates |
|  | Workforce arrangement: Division of duties among existing employees | Expansion of roles to include other tasks, adjustment of employee quarantine period depending on the situation, operation centered on middle managers, patience of non-infected staff members until the quarantine period of the COVID-19 infected staff ends, working with adjusted schedules, the handing of additional tasks related to infection control |
|  | Workforce retention: Efforts to retain staff through compensation and support | Motivational measures (additional financial compensation, allowing annual leave to be used while guaranteeing salary when the department was closed), persuasion and encouragement for staff retention |
|  | Minimization of employee exposure to infection risk | Persuading more staff to get vaccinations, conducting additional self-testing to avoid being an infection source, reducing unnecessary contact among existing staff, adjusting work schedules based on the identification of symptoms and close contact with individuals, tracking movements for infection prevention, restricting outings on the day off, providing and training on PPE |
| Safety | Changes in decisions regarding transfers | Retaining patients when designated hospitals run out of space |
|  | Infection prevention for patients and visitors | Disinfecting spaces, opening windows for ventilation, administering patient vaccinations, setting up isolation areas, strict control of outsiders’ access, pre-emptive PCR testing, efforts to stabilize family visits (contact-free visits and video calls through trial and error), efforts to separate clean and contaminated pathways |
| Continuity of essential services | Suspension of non-essential functions | Work pauses in traditional Korean medicine and rehabilitation therapy |
|  | Shifting perceptions of response planning | Specific planning after the occurrence of infected patients |
| Financial and supply resources | Bearing losses with constrained finances | Exceeded the contingency funds allocated for infection preparedness, striving for cost-saving efforts  Difficulty in proving losses to receive compensation |
|  | Efforts to secure uninterrupted supplies | Maintaining inventory levels at each LTCH level after recognizing the severity of the situation during a surge in infections |
|  |  |  |
